# Changes in inflammatory proteins following platelet transfusion in a neonatal population

**DOI:** 10.1038/s41390-023-02731-x

**Published:** 2023-07-13

**Authors:** Carmel Maria Moore, Daniel O’Reilly, Naomi McCallion, Anna E. Curley

**Affiliations:** 1 University College Dublin, Belfield, Dublin 4, Ireland; 2 National Maternity Hospital, Holles Street, Dublin 2, Ireland; 3 Rotunda Hospital, Parnell Square, Dublin 1, Ireland; 4 Royal College of Surgeons in Ireland, St Stephen’s Green, Dublin 2, Ireland

## Abstract

**Background:**

Studies have demonstrated increased morbidity and mortality with platelet transfusions in the neonatal period. Platelets are as important for host immunity and inflammation as for hemostasis. Increased inflammation may explain the dose-associated increase in mortality, bleeding, and lung disease.

**Objective:**

This study aims to assess if there are any changes in inflammatory cytokines post-platelet transfusion in babies in NICU.

**Methods:**

This prospective observational study recruited babies due to receive a non-emergency platelet transfusion. Dried whole blood samples were collected prior to and 2 h post-transfusion. Samples were processed using multiplex immunoassay to enable analysis of tiny blood volumes. Statistical analysis was performed using R.

**Results:**

Seventeen babies underwent 26 platelet transfusions across two centers. Median birthweight was 1545 g (535–3960 g) and median birth gestation was 31 weeks and 1 day (23 + 1 to 40 + 5). Median pre-transfusion platelet count was 19.5 × 10^9^/l. There was a significant increase in levels of CXCL5 (*p* < 0.001), CD40 (*p* = 0.001), and TGF-β (*p* = 0.001) in neonatal blood samples post-platelet transfusion in the study group.

**Conclusion:**

The increase in the cytokines CXCL5, CD40 and TGF-β after platelet transfusion in babies in NICU could potentiate existing inflammation, NEC, lung, or white matter injury. This could potentially explain long-term harm from platelet transfusion in babies.

**Impact:**

- There is a change in levels of immunomodulatory proteins CXCL5, CD40, and TGF-β after platelet transfusion in babies in NICU.
- Murine neonatal models have demonstrated an increase in cytokine levels after platelet transfusions. This is the first time that this has been demonstrated in human neonates.
- The increase in proinflammatory cytokines could potentially explain the long-term harm from platelet transfusion in babies, as they could potentiate existing inflammation, NEC, lung injury, or white matter injury.

## Introduction

Studies have demonstrated increased morbidity and mortality with platelet transfusions in the neonatal period.^1–3^ Although platelets comprise 10% of transfused blood components, they are responsible for 25–50% of serious adverse reactions^4^ including transfusion-related acute lung injury. Platelets play important roles in immunity, inflammation and angiogenesis, not merely in hemostasis.^5^ When platelets become activated, they degranulate, releasing a vast array of cytokines and chemokines, vasoactive agents, coagulation factors, adhesion molecules and growth factors that play a key role in modulating immune responses.^6^ Immunomodulatory effects may also enhance vascular permeability. Many neonates with severe thrombocytopenia are already critically ill and have a co-existing hyper-inflammatory state due to co-morbidities such as sepsis, necrotizing enterocolitis (NEC), and bronchopulmonary dysplasia (BPD). Recent studies on neonatal murine models have suggested that there is an increase in inflammation levels after platelet transfusions in neonatal mice.^7^ Murine studies have also demonstrated that transfused adult platelets are consumed faster than neonatal endogenous platelets in the early neonatal period, and platelet transfusion can enhance or attenuate the neonatal inflammatory response and mortality in a model of murine polymicrobial sepsis.^8^

There have not been any published studies to date investigating the inflammatory response to platelet transfusion in the neonatal population. This study aims to determine if there are changes in inflammatory protein expression after platelet transfusion in babies in the neonatal intensive care unit (NICU).

## Methods

This multicenter, prospective observational study was approved by the institutional Research Ethics Committees (National Maternity Hospital, Dublin 2, Ireland, EC 2020/28). Babies were recruited prior to commencement of the transfusion. Parents provided prospective informed consent. Two drops of whole blood were collected on a dried blood spot card prior to platelet transfusion. Two hours after the platelet transfusion, another two drops of the neonate’s blood were collected on a dried blood spot card. The cards were dried at room temperature and then refrigerated at 4°C until processing. Blood was collected via an indwelling arterial line when present, when venous access was being obtained for another reason or by capillary sampling. Blood spot sampling was used to minimize volumes of blood sampled for research. Samples were shipped at the same time and under the same conditions to the processing center.

Platelets transfused were sourced from one Blood Transfusion Service Laboratory (Irish Blood Transfusion Service, Dublin 8, Ireland), and were apheresis-derived. Platelets were irradiated and were not pathogen reduced. Platelets for neonatal use in this service are collected from donors providing at least two adult treatment doses of platelets in one apheresis session. They are tested including for bacteriology and released for use on the second day post-collection. Platelets are stored on agitator in the Blood Transfusion Service Laboratory and transferred to the hospital blood bank when requested, before release to the patient.

A high-throughput, multiplex immunoassay from Olink (Uppsala, Sweden) was chosen, as we aimed to minimize the blood volume required for testing due to the risk of phlebotomy-induced anemia in the NICU. This platform enables the analysis of 92 inflammation-related protein biomarkers across 96 samples simultaneously and can be performed in dried blood spot samples from neonates.^9^ Olink’s technology utilizes a proximity extension assay (PEA). PEA uses antibody pairs that are linked to oligonucleotides that have a slight affinity to each other (PEA probes). When target binding occurs, the PEA probes are brought in proximity and the two oligonucleotides are extended by DNA polymerase, forming a new sequence that acts as a unique surrogate marker for the specific antigen.^10^

The effect of long-term storage of these samples has been assessed,^9^ and in other studies, investigators have also looked at blood protein profiles in preterm babies using this method.^11,12^ We ran a five-sample pilot to ensure that our samples were being run under optimal conditions, guiding the sample dilution (1 in 4) to confirm that the data would be in the dynamic, linear range of the assay.

Statistical analysis was performed using R statistical software.^13^ Comparison of means was performed using a two-tailed paired *T*-test, as data were normalized to a log2 scale (Normalized protein eXpression (NPX)). As multiple pairwise comparisons were performed, *p* values were adjusted to control for the false discovery rate (FDR) using the Benjamini–Hochberg procedure using the ‘p.adjust’ package in R. Proteins which remained statistically significant following adjustment of *p* values were presented as boxplots using ‘ggplot2’ package in R.^14^

## Results

Seventeen babies had 26 platelet transfusions across the two centers. Pre-transfusion and post-transfusion samples were collected from all babies. Demographics are detailed in Table 1, and transfusion details are summarized in Table 2.

**Table 1:** Basic demographic information and diagnoses of included babies.

	Median	Range
Birthweight (g)	1545	535–3960
Gestation at birth (weeks+days)	31 + 1	23 + 1 to 40 + 5
First platelet count after birth (×10^9^/l)	124	20–246
Age at time of first transfusion (days)	3	1– 37
	Number	%
Female	8	47
Preterm	12	70.6
Antenatal diagnoses	Number	%
IUGR	2	11.8
Antepartum hemorrhage	4	23.6
Chorioamnionitis	2	11.8
Postnatal diagnoses	Number^a^	%^a^
Pulmonary hemorrhage	1	5.9
Subgaleal bleed	1	5.9
Bone marrow failure	1	5.9
Down syndrome	1	5.9
Noonan syndrome	1	5.9
Necrotizing enterocolitis	1	5.9
Congenital CMV	1	5.9
Sepsis	2	11.8
Clinical diagnosis DIC	3	17.6
Neonatal encephalopathy	6	35.3
Therapeutic hypothermia	5	29.4

^a^Some babies had more than one relevant postnatal diagnosis.

**Table 2:** Information about transfusions included.

	Median	Range
Pre-transfusion platelet count (×10^9^/l)	19.5	5 to 85
Transfusion duration (min)	60	30 to 60
Transfusion volume per kg (ml)	15.1	10.2 to 19.9
Platelet increment (where available) (×10^9^/l)	36.5	−13 to 101

**Fig. 1:**
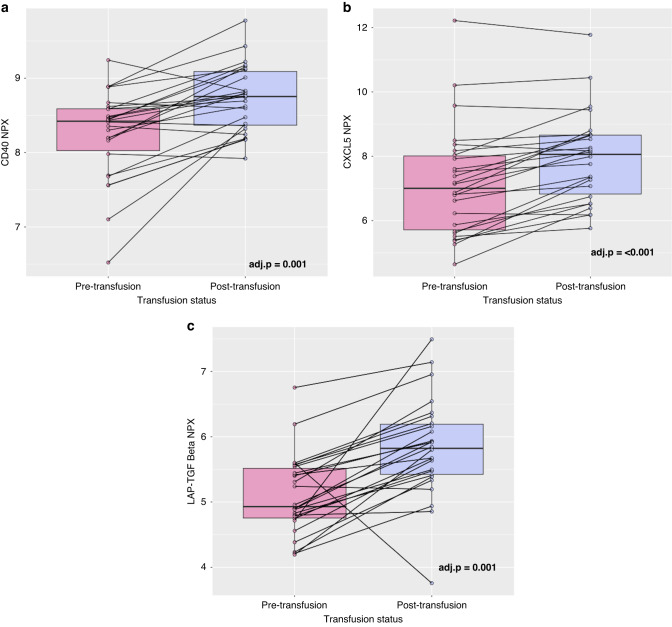
Differences in protein expression before and after platelet transfusion. **a** Dotplot demonstrating mean difference between CD40 protein expression (NPX) pre and post platelet transfusion. **b** Dotplot demonstrating mean difference between CXCL5 protein expression (NPX) pre and post platelet transfusion. **c** Dotplot demonstrating mean difference between LAP-TGF-ß pre and post platelet transfusion. Error bars represent standard error. Adj.*p* = Adjusted *p* values corrected for FDR using the Benjamini–Hochberg method.

Following comparison of pre- and post-transfusion samples, nine proteins were deemed statistically significant at a *p* value of <0.05: CD8A, Glial cell line-derived neurotrophic factor (GDNF), Latency associated peptide-transforming growth factor-ß (LAP-TGF-ß), Oncostatin M (OSM), Interleukin-10 Receptor Beta Unit (IL10RB), C-X-C motif Chemokine 5 (CXCL5), CD40, Sulfotransferase 1A1 (ST1A1) and macrophage colony-stimulating factor (CSF-1). The 95% confidence intervals and *p* values pre- and post-correction for FDR are available in Supplementary Table 1. Following correction for FDR as described above, three proteins remained statistically significant, CXCL5 (adjusted *p* ≤ 0.001, fold change (FC) = 0.76, 95% CI 0.47–1.05), LAP-TGF-ß (adjusted *p* = 0.001, FC = 0.72, 95% CI 0.42–1.02), CD40 (adjusted *p* = 0.001, FC = 0.48, 95% CI 0.2–0.6) (Fig. 1a–c).

## Discussion

There are multiple potential causes of platelet transfusion-induced harm. Neonates have shorter bleeding times despite the relative hyporeactivity and decreased adhesive capacity of their platelets.^15,16^ Transfusion of adult platelets to neonates could disturb this balance, potentially leading to increased thrombosis.^15^ The pathophysiology of potential damage from platelet transfusions may also be related to the hemodynamic effects of volume. Preterm infants are vulnerable to large changes in intravascular volume and blood pressure, and rapid infusion may make bleeding more likely in this vulnerable group. Of note, however, there was no increase in early bleeding noted in the PlaNeT-2/MATISSE trial between the two groups to suggest hemodynamic effect as a major factor in pathophysiology of bleeding.^2^ An inflammatory cause seems more likely to contribute to harm in the neonatal setting. This is the first neonatal study that has demonstrated changes in inflammatory proteins following platelet transfusion.

We know that in babies, there is a critical and synergistic interaction between infection/inflammation and hypoxia-ischemia.^17^ Inflammation and the production of systemic cytokines may also disturb already fragile cerebrovascular autoregulation, increasing the likelihood of brain injury.^18^ The significance of the increases in CXCL5, CD40 and TGF β seen in this study is that they all modulate existing inflammation, NEC, lung injury or white matter injury, which could potentially explain long-term harm from platelet transfusion in babies, through their release from platelets as thromboinflammatory modulators.^5^ CXCL5 (also known as ENA78) is a chemokine that induces neutrophil chemotaxis. It has been associated with NEC in neonatal^19,20^ and animal models.^21^ CXCL5 has also been associated with BPD in neonatal mice with NEC,^22^ and in non-NEC-related neonatal animal models of BPD.^23^ CXCL5 has also been associated with white matter injury in a neonatal rat model.^24^ Transforming growth factor-β is a growth factor that acts in a context-dependent manner on endothelial cells, monocytes, lymphocytes, natural killer cells and neutrophils, and affects multiple regulatory and inflammatory pathways to influence chemotaxis, activation, chemokine release and cell survival.^5^ Studies have suggested that TGF-β is associated with the development of BPD in neonatal models^25–28^ due to the stimulation of fibrosis. There is also evidence that TGF-β may be associated with the development of neonatal post-hemorrhagic hydrocephalus.^29^ However, higher TGF beta concentrations appear to correlate negatively with the development of NEC, where its immunomodulatory functions may be protective.^30,31^

CD40 upregulates leukocyte adhesion molecules and stimulates endothelial release of proinflammatory cytokines. CD40 is present in platelet transfusions in both the alpha granules and the platelet membranes, and it acts on the endothelial cell.^5^ Reviews have suggested an association between CD40 and retinopathy of prematurity,^32^ and evidence suggests that CD40 may be protective against NEC.^33^ Recent studies with murine models have demonstrated that platelet transfusions induce inflammation in neonatal mice without underlying inflammatory conditions.^7^

Whilst these changes may be simply due to an increase in absolute platelet counts following transfusion (particularly as sampling was performed using dried blood spots and not platelet-poor plasma), the lack of statistical change in other platelet-associated proteins including CCL3 and CXCL1 suggests that this represents a true modified response as opposed to bulk degranulation of an increased number of platelets. However, this study was designed to measure changes in proteins contained in whole blood, which includes plasma as well as intracellular proteins. It is not entirely possible to determine whether the post-transfusion increases in platelet-derived cytokines were due to the increased number of platelets in circulation or representative of immune response to platelet transfusion alone.

The limitations of this study are that it has small absolute numbers, and the statistical power is derived from perfectly paired data. The study population is heterogenous, with a wide range in gestational ages and different underlying disease processes causing thrombocytopenia and the requirement for platelet transfusion. There was also a short period between the pre- and post-transfusion sample, which may underestimate the true magnitude of post-transfusion changes in protein levels given the brief time allowed for translation to occur within this study design.^34^ This short time period did not enable assessment of any persisting changes in protein levels, which may also be important; however, the fold change in these cytokines seen in our study is significant, particularly given the short intervening period (2 h) between the pre-transfusion and post-transfusion samples.

## Conclusion

This study has demonstrated an increase in levels of CXCL5, CD40 and TGF β in blood samples following platelet transfusion supporting a potential role for inflammation in the pathophysiology of harm from platelet transfusion.

### Supplementary information

Supplementary Table 1

## Data Availability

The datasets generated and analyzed during the current study are available from the corresponding author on reasonable request.

## References

[1] Elgendy MM (2021). Platelet transfusion and outcomes of preterm infants: a cross-sectional study. Neonatology.

[2] Curley A (2019). Randomized trial of platelet-transfusion thresholds in neonates. N. Engl. J. Med..

[3] Kumar, J. et al. Platelet transfusion for PDA closure in preterm infants: a randomized controlled trial. *Pediatrics* **143**, e20182565 (2019). 10.1542/peds.2018-2565 30940676

[4] Garraud O (2016). Improving platelet transfusion safety: biomedical and technical considerations. Blood Transfus..

[5] McFadyen JD, Kaplan ZS (2015). Platelets are not just for clots. Transfus. Med. Rev..

[6] Stolla M (2015). Platelet transfusion – the new immunology of an old therapy. Front. Immunol..

[7] Davenport PE, Nolton E, Feldman H, Liu Z-J, Sola-Visner M (2021). Pro-inflammatory effects of platelet transfusions in newborn mice with and without underlying inflammation. Blood.

[8] Davenport PE (2021). Platelet transfusions can increase or decrease the inflammatory response and mortality in a murine model of neonatal polymicrobial sepsis. Blood.

[9] Björkesten J (2017). Stability of proteins in dried blood spot biobanks. Mol. Cell. Proteom..

[10] Assarsson E (2014). Homogenous 96-Plex pea immunoassay exhibiting high sensitivity, specificity, and excellent scalability. PLoS One.

[11] Zhong W (2021). Dramatic changes in blood protein levels during the first week of life in extremely preterm infants. Pediatr. Res..

[12] Olin A (2018). Stereotypic immune system development in newborn children. Cell.

[13] Team, R. C. *R: A Language and Environment for Statistical Computing*; https://www.R-project.org (2021).

[14] Wickham, H. *Ggplot2: Elegant Graphics for Data Analysis* (2016).

[15] Ferrer-Marin F, Stanworth S, Josephson C, Sola-Visner M (2013). Distinct differences in platelet production and function between neonates and adults: implications for platelet transfusion practice. Transfusion.

[16] Sola-Visner M (2012). Platelets in the neonatal period: developmental differences in platelet production, function, and hemostasis and the potential impact of therapies. Hematol. Am. Soc. Hematol. Educ. Program.

[17] Eklind S (2001). Bacterial endotoxin sensitizes the immature brain to hypoxic-ischaemic injury. Eur. J. Neurosci..

[18] Bassan H (2009). Intracranial hemorrhage in the preterm infant: understanding it, preventing it. Clin. Perinatol..

[19] Olaloye, O. O. et al. Cd16+Cd163+ monocytes traffic to sites of inflammation during necrotizing enterocolitis in premature infants. *J. Exp. Med.* **218**, e20200344 (2021). 10.1084/jem.20200344 PMC8289692 34269788

[20] Elfarargy MS (2019). Early biomarkers in neonatal necrotizing enterocolitis: a pilot study. J. Popul. Ther. Clin. Pharm..

[21] MohanKumar K (2012). Gut mucosal injury in neonates is marked by macrophage infiltration in contrast to pleomorphic infiltrates in adult: evidence from an animal model. Am. J. Physiol. Gastrointest. Liver Physiol..

[22] Jia H (2016). Pulmonary epithelial Tlr4 activation leads to lung injury in neonatal necrotizing enterocolitis. J. Immunol..

[23] Hirani, D. et al. Macrophage-derived Il-6 trans-signalling as a novel target in the pathogenesis of bronchopulmonary dysplasia. *Eur. Respir. J.* **59**, 2002248 (2022). 10.1183/13993003.02248-2020 PMC8850688 34446466

[24] Wang LY, Tu YF, Lin YC, Huang CC (2016). Cxcl5 signaling is a shared pathway of neuroinflammation and blood-brain barrier injury contributing to white matter injury in the immature brain. J. Neuroinflammation.

[25] Sakurai R, Li Y, Torday JS, Rehan VK (2011). Curcumin augments lung maturation, preventing neonatal lung injury by inhibiting TGF-Β signaling. Am. J. Physiol. Lung Cell. Mol. Physiol..

[26] Kunzmann S, Speer CP, Jobe AH, Kramer BW (2007). Antenatal inflammation induced TGF-Beta1 but suppressed CTGF in preterm lungs. Am. J. Physiol. Lung Cell. Mol. Physiol..

[27] Kwong KY (2006). Expression of transforming growth factor beta (TGF-B1) by human preterm lung inflammatory cells. Life Sci..

[28] Popova AP (2010). Autocrine production of TGF-Beta1 promotes myofibroblastic differentiation of neonatal lung mesenchymal stem cells. Am. J. Physiol. Lung Cell. Mol. Physiol..

[29] Cherian S, Whitelaw A, Thoresen M, Love S (2004). The pathogenesis of neonatal post-hemorrhagic hydrocephalus. Brain Pathol..

[30] Pang Y, Du X, Xu X, Wang M, Li Z (2018). Impairment of regulatory T cells in patients with neonatal necrotizing enterocolitis. Int. Immunopharmacol..

[31] Maheshwari A (2011). TGF-Β2 suppresses macrophage cytokine production and mucosal inflammatory responses in the developing intestine. Gastroenterology.

[32] Aouiss A, Anka Idrissi D, Kabine M, Zaid Y (2019). Update of inflammatory proliferative retinopathy: ischemia, hypoxia and angiogenesis. Curr. Res. Transl. Med..

[33] Xu J (2011). Ileal immune dysregulation in necrotizing enterocolitis: role of CD40/CD40L in the pathogenesis of disease. J. Pediatr. Gastroenterol. Nutr..

[34] Liu C (2021). Cytokines: from clinical significance to quantification. Adv. Sci..

